# Optimal Implementation Parameters of a Nonlinear Electrical Impedance Tomography Method Using the Complete Electrode Model

**DOI:** 10.3390/s22176667

**Published:** 2022-09-03

**Authors:** Jeongwoo Park, Jun Won Kang, Eunsoo Choi

**Affiliations:** Department of Civil and Environmental Engineering, Hongik University, Seoul 04066, Korea

**Keywords:** electrical impedance tomography, inverse problem, complete electrode model, optimal implementation parameters, partial-differential-equation-constrained optimization

## Abstract

This study discusses a nonlinear electrical impedance tomography (EIT) technique under different analysis conditions to propose its optimal implementation parameters. The forward problem for calculating electric potential is defined by the complete electrode model. The inverse problem for reconstructing the target electrical conductivity profile is presented based on a partial-differential-equation-constrained optimization approach. The electrical conductivity profile is iteratively updated by solving the Karush–Kuhn–Tucker optimality conditions and using the conjugate gradient method with an inexact line search. Various analysis conditions such as regularization scheme, number of electrodes, current input patterns, and electrode arrangement were set differently, and the corresponding results were compared. It was found from this study that the proposed EIT method yielded appropriate inversion results with various parameter settings, and the optimal implementation parameters of the EIT method are presented. This study is expected to expand the utility and applicability of EIT for the non-destructive evaluation of structures.

## 1. Introduction

Electrical impedance tomography (EIT) is a non-destructive evaluation method through which the electrical properties of part of a structure are determined using measured data from surface electrodes. This method is highly applicable in medical imaging, industrial process monitoring, and geotechnical site characterization because of its ease of use in field experimentation, economic feasibility, and superior ability to penetrate the target. For example, the EIT method coupled with convolutional neural networks has been explored for reconstructing human organ boundaries [1], and EIT using an optimal control theory has been developed for pathological diagnoses such as cancer detection [2]. In industrial processes, EIT can be a useful tool to monitor the mixing process of chemical materials [3,4] and evaluate the dredging process’s condition in real-time by monitoring the material flowing through the dredging pipe [5,6]. Recent applications of EIT in civil engineering include the characterization of layered soils [7], crack detection in pipes buried in the ground [8], and ground contamination monitoring for remediation strategies [9].

EIT has many advantages; however, it requires improvements in its mathematical modeling, numerical analysis, and implementation techniques to increase the accuracy of solutions and to broaden its application scope to further extensive fields. Previous studies on EIT suggest that the quality of the inverse tomographic images obtained using measured electric potentials is sensitive to electrode arrangement and current input patterns [10,11,12,13,14,15,16]. Graham et al. [11] presented seven electrode placement configurations (planar, planar-offset, planar-opposite, zigzag, zigzag-offset, zigzag-opposite, square) in which the electrodes were arranged in two parallel planes of eight electrodes each, with electrodes equispaced around a medium. These configurations were applied to three-dimensional (3D) EIT, and the inversion results concerning the electrode arrangements were compared. Consequently, not one electrode placement configuration offered a good improvement over the others under ideal conditions. However, when noise and electrode placement errors are considered, the choice of electrode placement becomes important, and under that condition, planar electrode placement has the best overall performance. Schullcke et al. [12] evaluated the effect of different numbers of electrodes used for current injection and voltage measurements on the reconstructed two-dimensional shape of the lungs. The number of electrodes was varied systematically in steps of four, from n=8 to n=32. According to the research results, the increased number of electrodes does not necessarily increase the image quality. The reconstructions made with 16 electrodes preserved the best quality.

Examples of current input patterns commonly used in EIT include the adjacent drive pattern [13], opposite or polar drive patterns [14], and trigonometric patterns [15]. The adjacent drive pattern is sensitive to conductivity contrasts near the boundary and insensitive to central contrasts. Furthermore, it is imperative to measurement errors and noise. The opposite or polar drive pattern is less sensitive to conductivity changes at the boundary in relation to the adjacent method, as the current travels with higher uniformity through the imaged body [16]. In the trigonometric pattern, the current is injected on all electrodes, and voltages are measured at all electrodes. This pattern results in more stable and accurate reconstruction than the adjacent current pattern. However, current drivers are required for each electrode to be injected, and the unknown contact impedance will affect the reconstruction [16,17,18].

Based on the previous developments, this study proposes several implementation parameters and analysis conditions for EIT, such as the number of electrodes, current input pattern, and electrode arrangement. The proposed parameters and analysis conditions are applied to the EIT procedure described in a recent study [19,20]. The EIT method is a nonlinear inversion based on a partial-differential-equation (PDE)-constrained optimization approach. By applying various implementation parameters, this study presents the optimal parameters of the EIT method that minimize relative $L^{2}$-error or relative misfit. It also shows how much the quality of the reconstructed electrical conductivity profile using the optimal implementation parameters is improved in relation to the results using a conventional parameter set.

The remainder of this paper is organized as follows: In Section 2, the forward problem based on the complete electrode model (CEM) is presented. For verification, the forward solution was compared with that obtained using ANSYS Mechanical APDL [21]. In Section 3, the inverse medium problem is described, which derives the optimal solution using the Lagrangian functional and the first-order optimality conditions. The inversion process updates the electrical conductivity profile iteratively using a conjugate gradient method with an inexact line search. In Section 4, various numerical examples are described, wherein the regularization scheme, number of electrodes, current input pattern, and electrode arrangement are set differently. Optimal implementation parameters are suggested for the best reconstruction of a layered profile. Section 5 presents the conclusions of the study.

## 2. The Forward Problem

### 2.1. Mathematical Method

**Figure 1:**
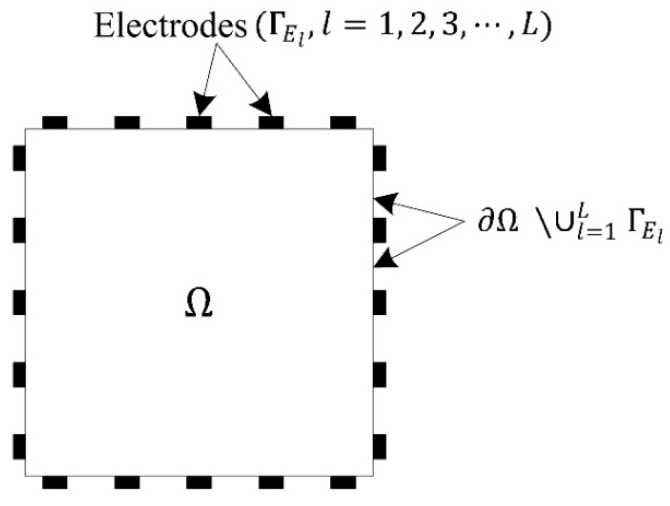
Configuration of a two-dimensional square domain with surface electrodes.

The CEM is a mathematical model for resolving the electrostatic forward problem for electric potential. It considers current loss that occurs when the current flows to a low-impedance material through an electrode and the voltage drop due to the contact impedance between the structural surface and electrode [22,23]. Therefore, the error between the calculated electric potential and experimentally obtained data is smaller than that computed using other models, such as a point electrode model. Figure 1 shows the configuration of a two-dimensional (2D) square domain with electrodes on its surface.

The CEM for calculating the electric potential due to the current input can be expressed as a boundary value problem, as follows:

(1)∇·(σ∇u)=0,x∈Ω,(2)u+zlσ∂u∂n=Ul,l=1, 2, ⋯, L, x∈ΓEl,(3)∫ΓElσ∂u∂ndΓ=Ill=1, 2, ⋯, L, x∈ΓEl,(4)σ∂u∂n=0,x∈∂Ω \∪l=1L°ΓEl,

where Ω denotes structural domain, u is the scalar-valued electric potential to be calculated, σ is electrical conductivity, n is the outward unit normal to the boundary ∂Ω, $\Gamma_{E_{l}}$ is the lth electrode boundary, $z_{l}$ is the contact impedance of $E_{l}$, $I_{l}$ is the injected current at $E_{l}$, $U_{l}$ is the electric potential at $E_{l}$ to be calculated, and L is the number of electrodes. Equation (1) is a Laplace equation for the electric potential u(x). Equation (2) is a Robin-type boundary condition describing the electric potential at $\Gamma_{E_{l}}$, and Equations (3) and (4) are Neumann boundary conditions for u(x). To ensure the existence and uniqueness of the solution, the following continuity condition,

(5) $$\overset{L}{\underset{l=1}{\sum }}I_{l}=0,$$

is added to the model. For setting the reference point of the electric potential,

(6) $$\overset{L}{\underset{l=1}{\sum }}U_{l}=0$$

must be satisfied. For the variational form of the boundary value problem, Equation (1) is multiplied by a test function $v\left(x\right)\in H^{1}\left(\Omega \right)$, and then integrated over the domain Ω using boundary conditions (2), (3), and (4). Meanwhile, Equation (2) is integrated over $\Gamma_{E_{l}}$, multiplied by a test value $V_{l}$, and then summed for all electrodes. Adding the two equations results in a variational form, expressed as follows [24]:

(7) $$\int_{\Omega }^{}\sigma \nabla u\;\cdot \nabla vd\Omega +\overset{L}{\underset{l=1}{\sum }}\frac{1}{z_{l}}\int_{\Gamma_{E_{l}}}^{}\left(u-U_{l}\right)\left(v-V_{l}\right)d\Gamma =\overset{L}{\underset{l=1}{\sum }}I_{l}V_{l}.$$

Introducing finite element approximations to the electric potential u(x) and the test function v(x). results in a linear system of equations, where the electric potential u at each node and the electrode potential $U_{l}$ can be calculated. The stiffness matrix and the right-hand-side vector of the linear system can be found in [19,20].

### 2.2. Setting for Numerical Analysis

**Figure 2:**
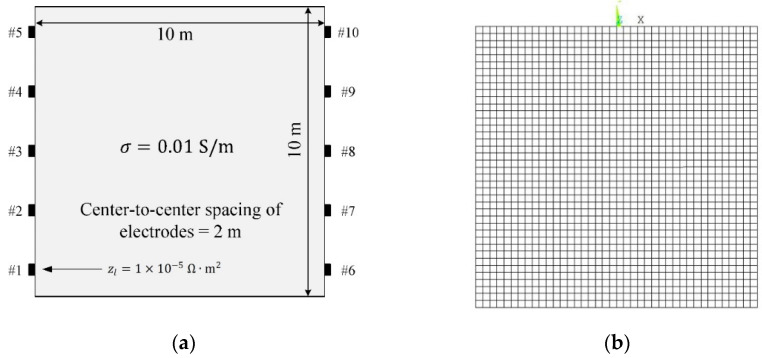
(**a**) Configuration of a homogeneous square domain and (**b**) finite element mesh with eight-node quadrilateral elements.

The forward CEM solution is validated by comparing it to the solution obtained by ANSYS Mechanical APDL. Figure 2a shows the configuration of a homogeneous square domain with a side length of 10 m. The electrical conductivity of the domain is 0.01 S/cm. A total of 10 electrodes are distributed equally on the left and right sides, as shown in the figure. Figure 2b shows a finite element model consisting of 1600 eight-node square elements with sides of 0.25 m. The length of one electrode is 0.25 m, equal to the size of the finite element. A current of 0.1 A is input into the electrodes placed on the left side of the domain, and it flows out through the electrodes placed on the right side. The contact impedance of the electrodes is $1\times 10^{-5}\;\Omega \cdot m^{2}$.

### 2.3. Results of the Validation

**Figure 3:**
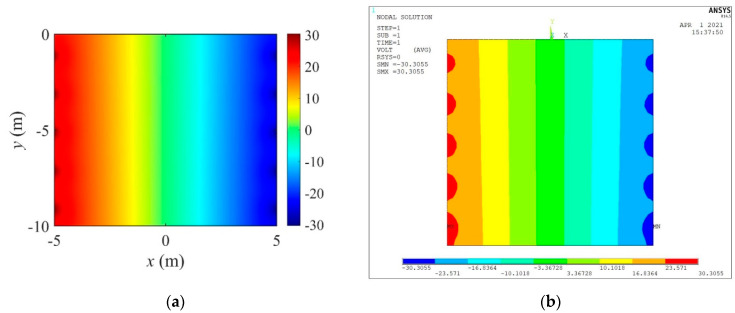
Electric potential distribution calculated using CEM and ANSYS APDL. (**a**) CEM. (**b**) ANSYS APDL.

**Figure 4:**
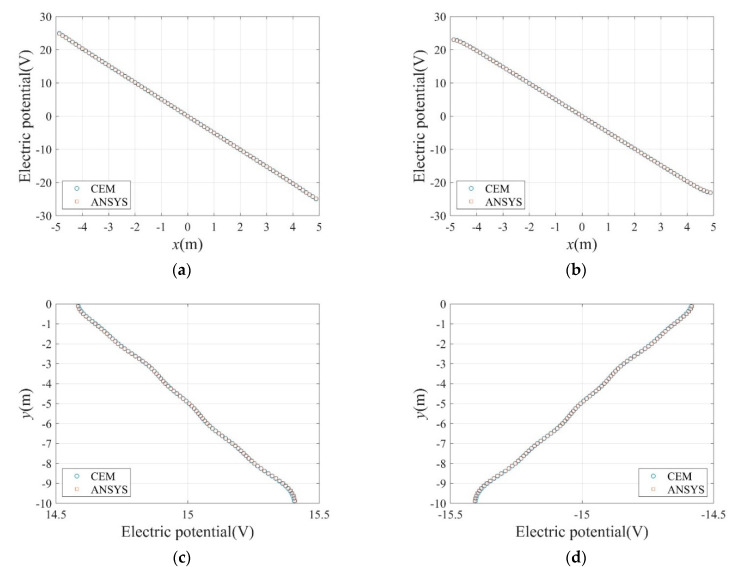
Calculated electric potential values along particular lines in domain. (**a**)  y=−7.5 m. (**b**)  y=−2.5 m. (**c**) x=−3.0 m. (**d**)  x=3.0 m.

Figure 3a,b show the distribution of the electric potential calculated by the CEM and ANSYS APDL, respectively, owing to the current input. In Figure 4, calculated electric potential values using the CEM and ANSYS APDL at nodes on (a) y=−7.5 m, (b) y=−2.5 m, (c) x=−3.0 m, and (d) x=3.0 m are compared. It can be observed that the solutions obtained by the two different solvers are similar at all positions.

## 3. The Inverse Problem

### 3.1. PDE-Constrained Optimization

The inverse medium problem for reconstructing the electrical conductivity profile of a structure using measured electric potential data at surface electrodes can be presented as the following PDE-constrained optimization problem:

(8) $$\underset{\sigma \left(x\right)}{\min}J≔\frac{1}{2}\overset{L}{\underset{l=1}{\sum }}\int_{\Gamma_{E_{l}}}^{}{\left(U_{l}-U_{l}^{m}\right)}^{2}d\Gamma +\gamma \left(\sigma \right).$$

The objective functional J comprises a misfit functional and a regularization term. The misfit functional is expressed as the sum of the squared differences of the calculated electric potential $U_{l}$ and the measured electric potential $U_{l}^{m}$ at electrode $E_{l}$. This optimization problem is constrained by Equation (1), which is the governing equation of the CEM, and boundary conditions (2)–(4). To relieve the ill-posedness present in such an inverse problem, the regularization term γ(σ) for the electrical conductivity σ is included in the objective functional J.

### 3.2. Regularization Schemes

In this study, Tikhonov (TN) [25] and total variation (TV) [26] regularization schemes are used to investigate the regularization effect. For TN regularization, the regularization term γ(σ) can be expressed by Equation (9):

(9) $$\gamma^{TN}\left(\sigma \right)=\frac{1}{2}R_{\sigma }\int_{\Omega }^{}\nabla \sigma \cdot \nabla \sigma d\Omega \;,$$

For TV regularization, γ(σ) can be expressed by Equation (10):

(10) $$\gamma^{TV}\left(\sigma \right)=R_{\sigma }\int_{\Omega }^{}{\left(\nabla \sigma \cdot \nabla \sigma +\beta \right)}^{\frac{1}{2}}d\Omega \;,$$

where $R_{\sigma }$ is a regularization factor that controls the penalty for the spatial variation of electrical conductivity σ(x). In Equation (10), a small parameter β is included to make $\gamma^{\mathrm{TV}}\left(\sigma \right)$ differentiable when ∇σ=0. Generally, it is expected that TN regularization would be suitable for reconstructing a smooth target profile. On the other hand, TV regularization is expected to perform better when reconstructing a sharply varying target profile.

### 3.3. First-Order Optimality Conditions

The Lagrange multiplier method was used to convert the PDE-constrained optimization problem written in Equation (8) into an unconstrained optimization problem. The objective functional J can be augmented using Equations (1) and (2) to construct the Lagrangian functional ℒ:

(11) $$\begin{array}{ll} ℒ(u,\;U_{l},\;w,\;W_{l},\;\sigma ) & =\frac{1}{2}\overset{L}{\underset{l=1}{\sum }}\int_{\Gamma_{E_{l}}}^{}{\left(U_{l}-U_{l}^{m}\right)}^{2}d\Gamma +\gamma \left(\sigma \right)+\int_{\Omega }^{}w\nabla \cdot \left(\sigma \nabla u\right)d\Omega \\ & +\overset{L}{\underset{l=1}{\sum }}\int_{\Gamma_{E_{l}}}^{}W_{l}\left(\sigma \frac{\partial u}{\partial n}-I_{l}\right)d\Gamma , \end{array}$$

where w and $W_{l}$ are Lagrange multipliers multiplied to the left-hand terms of the governing equation and boundary conditions, respectively. The electrical conductivity σ(x) that minimizes the Lagrangian ℒ is the solution to the inverse problem. For the optimal solution to this problem, the first-order optimality conditions of the Lagrangian are enforced. In other words, the first variation of ℒ with respect to adjoint variables w and $W_{l}$, state variables u and $U_{l}$, and control variable σ is enforced to vanish. There results the state problem for u and $U_{l}$, the adjoint problem for w and $W_{l}$, and the control problem for σ, respectively. Solving the three problems simultaneously in the reduced space of the control variable yields the optimal solution of the material profile σ(x).

#### 3.3.1. First Optimality Condition: State Problem

The state equation and the corresponding boundary conditions can be obtained from the stationarity requirement that the first variation of the Lagrangian with respect to adjoint variables w and $W_{l}$ must be 0 $(\delta_{w}ℒ=0,\;\delta_{W_{l}}ℒ=0,\;l=1,2,\cdots L$). The derived state problem is identical to the forward problem in Equations (1)–(4).

#### 3.3.2. Second Optimality Condition: Adjoint Problem

The adjoint equation and the corresponding boundary conditions can be obtained from the stationarity requirement that the first variation of the Lagrangian with respect to state variables u and $U_{l}$ must be 0 ($\delta_{u}ℒ=0,\delta_{U_{l}}ℒ=0,\;l=1,2,\cdots L$). The derived adjoint problem can be described as follows [19,20]:

(12)∇·(σ∇w)=0,x∈Ω,(13)w+zlσ∂w∂n=−Wl,l=1, 2, ⋯, L,x∈ΓEl,(14)σ∂w∂n=Ul−Ulm,l=1, 2, ⋯, L,x∈ΓEl,(15)σ∂w∂n=0,x∈∂Ω \∪l=1L ΓEl.

Equation (12) is the governing equation for the adjoint variable w(x). It has a differential operator similar to (1), the state equation. Equations (13)–(15) are the boundary conditions of the adjoint problem. Equation (14) indicates the source of the adjoint problem, which depends on the misfit of the electric potential at electrodes. The adjoint problem can also be solved by the finite element method in a manner similar to the state problem [20].

#### 3.3.3. Third Optimality Condition: Control Problem

The control equation and the corresponding boundary conditions can be obtained from the stationarity requirement that the first variation of the Lagrangian with respect to the control variable σ must be 0 ($\delta_{\sigma }ℒ=0$). The derived control problem can be described as follows [19,20]:

(16)−RσΔσ−∇w·∇u=0,x∈Ω,(17)Rσ∂σ∂n+w∂u∂n+Wl∂u∂n=0,l=1, 2, ⋯, L,x∈ΓEl,(18)Rσ∂σ∂n+w∂u∂n=0,x∈∂Ω \∪l=1L ΓEl.

In deriving Equation (16), the TN regularization scheme was used. If the TV scheme were used instead, Equation (16) could be replaced by

(19) $$\begin{array}{cc} -R_{\sigma }{\left(\nabla \sigma \cdot \nabla \sigma +\beta \right)}^{-\frac{3}{2}}\;\left[\left(\nabla \sigma \cdot \nabla \sigma +\beta \right)\Delta \sigma -\nabla \sigma \cdot \left(H\nabla \sigma \right)\right]-\nabla w\cdot \nabla u=0, & x\in \Omega , \end{array}$$

where H is the Hessian matrix of σ(x). It is different from the Hessian for the Gauss–Newton inversion, which consists of the second Fréchet derivatives of the Lagrangian [27,28,29]. The solution σ(x) of the control problem can be calculated once the state and adjoint solutions u, w, and $W_{l}$ are obtained. The state, adjoint, and control problems derived from the first-order optimality conditions of the Lagrangian indicate the Karush–Kuhn–Tucker (KKT) conditions for this optimization problem.

### 3.4. Material Property Update

The first and second optimality conditions are satisfied by solving the state and adjoint problems, respectively. Because only the true profile of σ(x) exactly satisfies the control problem, the material profile σ(x) must be updated to satisfy the third optimality condition. The procedure of updating the control variable σ(x) using the state and adjoint solutions is as follows:

1. Assume the initial electrical conductivity profile of a structure to be investigated, then calculate the electric potential u and $U_{l}$ due to the current input through the surface electrodes.
2. Calculate the adjoint solutions w and $W_{l}$ using the state solution $U_{l}$.
3. Using the state and adjoint solutions, calculate the gradient of the Lagrangian with respect to the control variable σ, as follows:
  (20) $$g_{\sigma }\equiv \nabla_{\sigma }ℒ=-R_{\sigma }\Delta \sigma -\nabla w\cdot \nabla u.$$
  In Equation (20), the TN regularization scheme was used. If the TV scheme were assumed, then the Lagrangian gradient for σ would be
  (21) $$g_{\sigma }\equiv \nabla_{\sigma }ℒ=-R_{\sigma }{\left(\nabla \sigma \cdot \nabla \sigma +\beta \right)}^{-\frac{3}{2}}\;\left[\left(\nabla \sigma \cdot \nabla \sigma +\beta \right)\Delta \sigma -\nabla \sigma \cdot \left(H\nabla \sigma \right)\right]-\nabla w\cdot \nabla u.$$
4. Update the electrical conductivity at each node using a line search method. Equations (17) and (18) are not precisely enforced in updating the electrical conductivity at boundaries since they are complicated to implement. Instead, one can enforce that the normal derivative of σ(x) be zero along the boundary for computational simplicity.

### 3.5. Conjugate Gradient Method with an Inexact Line Search

The search direction for the optimal solution of the control variable σ is determined using the Fl$\overset{´}{e}$tcher–Reeves conjugate gradient method with an inexact line search. Let $g_{k}$ denote the discrete reduced gradient at the kth inversion iteration.

(22) $$g_{k}={\left(\nabla_{\sigma }ℒ\right)}_{k}.$$

Thereafter, the electrical conductivity vector $\sigma_{k}$ comprising nodal values of σ is updated via

(23) $$\sigma_{k+1}=\sigma_{k}+\alpha d_{k},$$

where $d_{k}$ is the search direction vector at $\sigma_{k}$, and α. is the step length in the direction of $d_{k}$. The step length can be determined by a backtracking algorithm in Table 1 [30]. In this work, $\bar{\rho }=0.5$ was used.

### 3.6. Regularization Factor Continuation Scheme

The choice of regularization factor $R_{\sigma }$ in Equations (20) and (21) considerably affects the reconstruction of the electrical conductivity profile because it controls the amount of imposed penalty on high-frequency oscillations of the material properties. In this study, a regularization factor continuation scheme [30,31,32] was used to determine the optimal regularization factor at each inversion iteration. The reduced gradients in Equations (20) and (21) can be rewritten as

(24) $$\nabla_{\sigma }ℒ=R_{\sigma }\left(\nabla_{\sigma }J_{r}\right)+\nabla_{\sigma }J_{m},$$

where $R_{\sigma }\nabla_{\sigma }J_{r}$ denotes the gradient of the regularization functional and $\nabla_{\sigma }J_{m}$ the gradient of the misfit functional. In the case of the TN regularization,

(25) $$\nabla_{\sigma }J_{r}=-∆\sigma ,$$

(26) $$\nabla_{\sigma }J_{m}=-\nabla w\cdot \nabla u.$$

If the TV regularization scheme were used,

(27) $$\nabla_{\sigma }J_{r}=-{\left(\nabla \sigma \cdot \nabla \sigma +\beta \right)}^{-\frac{3}{2}}\;\left[\left(\nabla \sigma \cdot \nabla \sigma +\beta \right)∆\sigma -\nabla \sigma \cdot \left(H\nabla \sigma \right)\right],$$

(28) $$\nabla_{\sigma }J_{m}=-\nabla w\cdot \nabla u.$$

The first term of Equation (24), $R_{\sigma }(\nabla_{\sigma }J_{r})$, penalizes spatial oscillations in the reconstructed profile, such that a higher $R_{\sigma }$ results in a smoother reconstructed profile. A balance between these two terms can be imposed using

(29) $$R_{\sigma }\left|\nabla_{\sigma }J_{r}\right|<\left|\nabla_{\sigma }J_{m}\right|\;⟹\;R_{\sigma }<\frac{\left|\nabla_{\sigma }J_{m}\right|}{\left|\nabla_{\sigma }J_{r}\right|}.$$

Therefore, $R_{\sigma }$ can be calculated at each iteration as

(30) $$R_{\sigma }=\varepsilon \frac{\left|\nabla_{\sigma }J_{m}\right|}{\left|\nabla_{\sigma }J_{r}\right|},\;\;\;\left(0\leq \varepsilon \leq 1\right),$$

where ε is a weight factor, which plays the role of regularization effect controller. ε=1 results in the maximum regularization effect, and ε=0 indicates no regularization.

## 4. Numerical Studies for Optimal EIT Parameters

Consider a square domain with a side length of 10 m, as shown in Figure 1, surrounded by electrodes with a contact impedance of $1\times 10^{-5}\;\Omega \cdot m^{2}$. The initial value of the regularization factor $R_{\sigma }$ is 1.0, and the weight factor ε for the regularization factor continuation scheme is set to 0.5. The parameter β for TV regularization is assumed to be $\beta =1\times 10^{-6}$.

This study compares the inversion results according to the change in various implementation parameters using a response misfit and a relative $L^{2}$-error. The response misfit ${ℱ}_{m}$, as part of the objective functional J in Equation (8), can be written as

(31) $${ℱ}_{m}=\frac{1}{2}\overset{L}{\underset{l=1}{\sum }}\int_{\Gamma_{E_{l}}}^{}{\left(U_{l}-U_{l}^{m}\right)}^{2}d\Gamma .$$

The relative $L^{2}$-error of electrical conductivity, ${\left\|E\right\|}_{L^{2}}$ can be written as

(32) $${\left\|E\right\|}_{L^{2}}=\frac{1}{A}\int_{\Omega }^{}\frac{{\left(\sigma_{inv}-\sigma_{tg}\right)}^{2}}{\sigma_{tg}^{2}}\;d\Omega ,$$

where A is the total area of the domain, $\sigma_{tg}$ is the target electrical conductivity, and $\sigma_{inv}$ is the reconstructed electrical conductivity.

### 4.1. Regularization Effect

**Figure 5:**
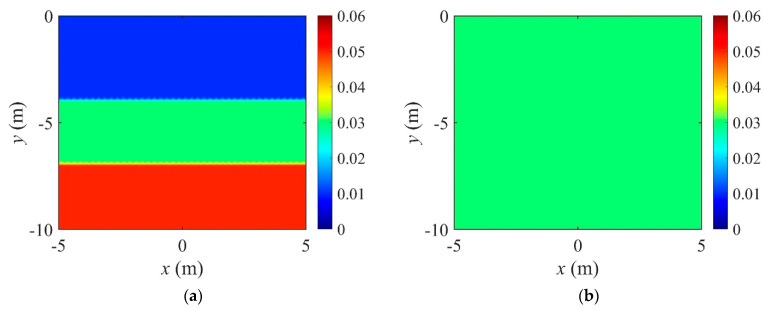
Target electrical conductivity profile with three layers and the initial guess. (**a**) Target. (**b**) Initial guess.

For evaluating the regularization effect on the EIT, the TN and TV regularization schemes are explored in the inversion for a three-layer heterogeneous medium. Figure 5 shows the target electrical conductivity profile with three layers and an initial guess for inversion. The target values of the electrical conductivity are 0.01 S/m for y≥−4 m, 0.03 S/m for −7 m≤y<−4 m, and 0.05 S/m for y<−7 m. The values are typical of air-dried concrete materials in various conditions [33]. Examples of the profile heterogeneity in Figure 5 include fiber-reinforced composite sandwich plates and concrete specimens under curing. The initial guess for the inversion $\sigma_{\mathrm{ini}}=0.03$ S/m. 40 electrodes are arranged on all sides of the square medium with equal spacing; thereafter, the current is injected into the electrodes attached on the top and left sides of the structure, and then flows out through the electrodes on the right and bottom sides. The magnitude of the current is uniform at 0.1 A.

**Figure 6:**
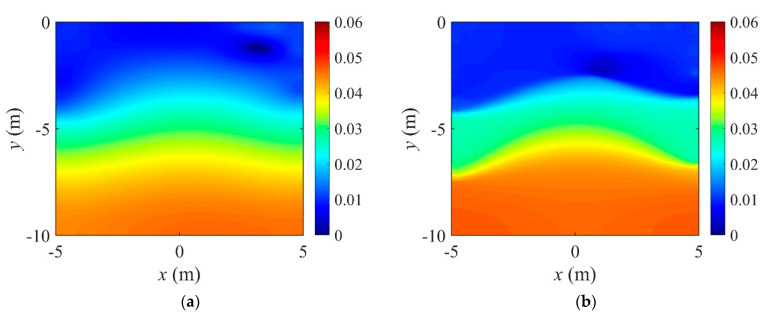
Inversion results of a three-layer electrical conductivity profile using TN and TV regularization schemes. (**a**) TN. (**b**) TV.

**Figure 7:**
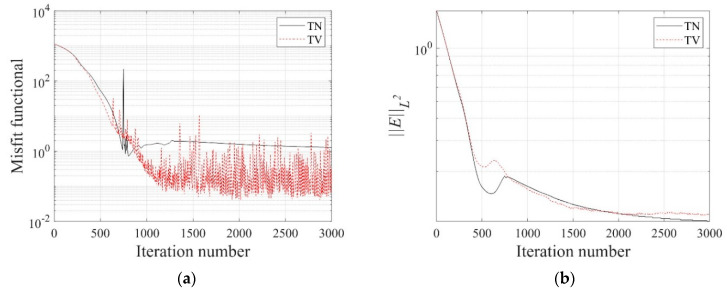
Variation of response misfit and the relative $L^{2}$-error during the inversion using TN and TV regularization schemes. (**a**) Misfit variation. (**b**) Relative $L^{2}$-error.

**Figure 8:**
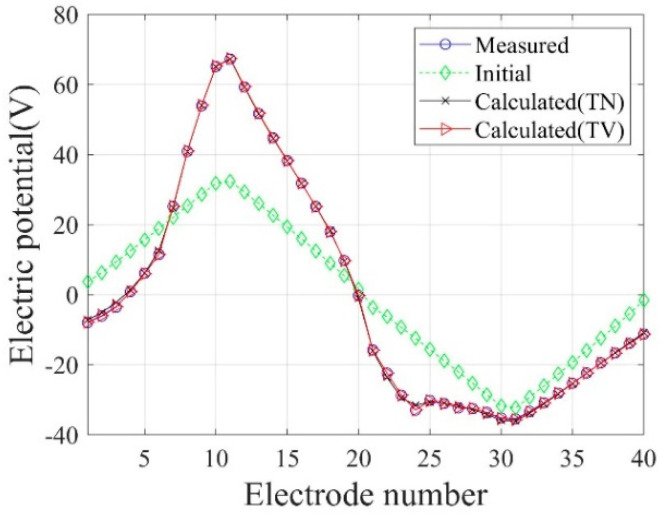
Measured, initial, and calculated electric potentials in the inversion using TN and TV regularization schemes.

**Figure 9:**
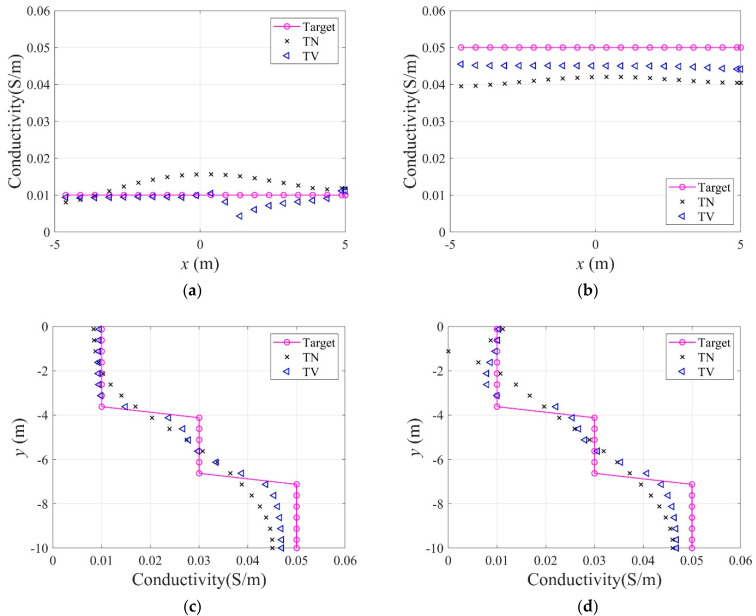
Reconstructed conductivity profiles in the horizontal and vertical directions. (**a**)  y=−2.5 m. (**b**)  y=−7.5 m. (**c**) x=−3.0 m. (**d**)  x=3.0 m.

Figure 6 shows the inversion results of the three-layer electrical conductivity profile at 3000 iterations using TN and TV regularization schemes. When the TV scheme was used, the target profile was reconstructed clearly and stably, especially at the interface of the layers. This shows that the TV scheme performs well in the EIT framework when reconstructing a sharply varying profile. Figure 7 shows the response misfit and the relative $L^{2}$-error ${\left\|E\right\|}_{L^{2}}$ against iteration numbers. The misfit is reduced by 99.8% from its initial value for the TN scheme, and by 99.9% for the TV at 1500 iterations. Figure 8 shows the measured, initial, and calculated electric potentials in the inversion. After the inversion, the calculated potential values nearly coincide with the measured values, indicating the successful reconstruction of the target profile. Figure 9 shows the target and reconstructed conductivity profiles at y=−7.5 m, y=−2.5 m, x=−3.0 m, and x=3.0 m in the domain. The results show that the recovered profile captures the variation of the target conductivity values in both directions.

**Figure 10:**
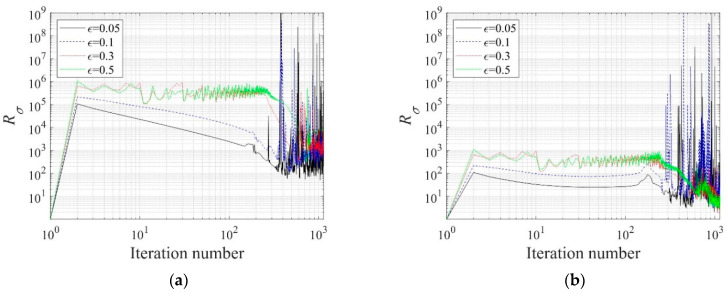
Regularization factor variation during the inversion using the regularization factor continuation scheme. (**a**) TN. (**b**) TV.

As mentioned in Section 3.6, the regularization factor $R_{\sigma }$ considerably affects the reconstruction of the electrical conductivity profile. Figure 10 shows the change in the regularization factor during the inversion using the regularization factor continuation scheme. The value of the weight factor ε was assumed to be 0.5, 0.3, 0.1, and 0.05. The higher the weight factor ε, the larger the $R_{\sigma }$ value. It can also be seen that the value of $R_{\sigma }$ fluctuates significantly in the latter part of the inversion as the inverted profile approaches the target.

**Figure 11:**
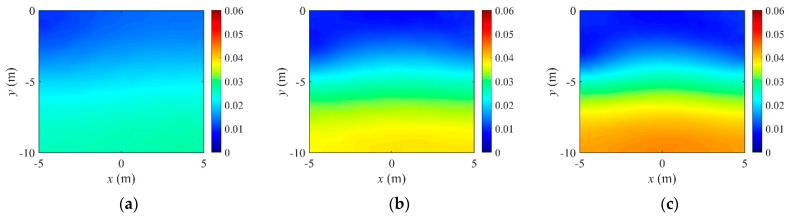
Inversion for a three-layer profile using fixed regularization factors for TN regularization. (**a**) TN, $R_{\sigma }=9.0\times 10^{5}$. (**b**) TN, $R_{\sigma }=8.0\times 10^{4}$. (**c**) TN, $R_{\sigma }=2.3\times 10^{4}$.

**Figure 12:**
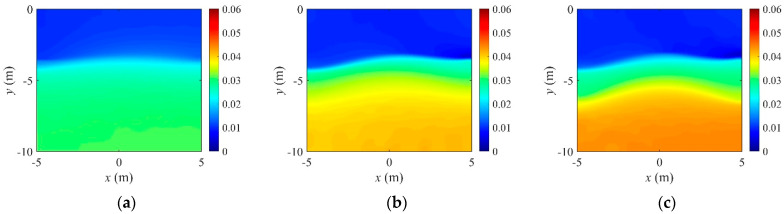
Inversion for a three-layer profile using fixed regularization factors for TV regularization. (**a**) TV, $R_{\sigma }=9.4\times 10^{2}$. (**b**) TV, $R_{\sigma }=9.5\times 10^{1}$. (**c**) TV, $R_{\sigma }=3.3\times 10^{1}$.

**Figure 13:**
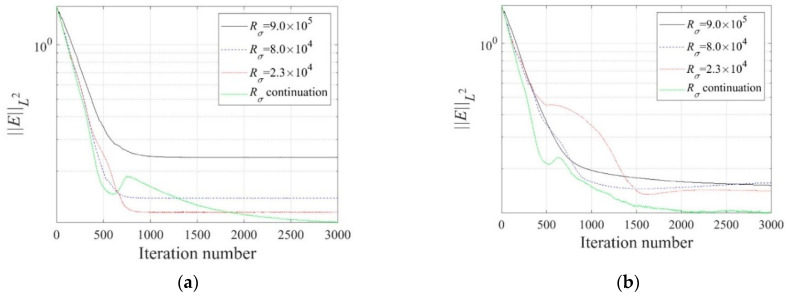
Variation of ${\left\|E\right\|}_{L^{2}}$ to iteration numbers for fixed and continuous regularization factor schemes. (**a**) TN. (**b**) TV.

For demonstrating the effectiveness of the regularization factor continuation scheme, the inversion tried fixed regularization factors in the same setting. Figure 11 and Figure 12 show the reconstructed three-layer electrical conductivity profiles using different fixed regularization factors. The layer interfaces are not properly recovered when $R_{\sigma }$ is large, but the stratum is better reconstructed when the factor is small. Again, the TV scheme yielded sharper profile reconstruction than the TN. Compared to using fixed regularization factors, the continuation scheme determines the regularization factor adaptively at each inversion iteration, making it possible to skip multiple inversion attempts to find the optimal fixed regularization factor. Figure 13 shows the variation of the relative $L^{2}$-error in Equation (32) to iteration numbers. In the case of the continuous regularization factor, the relative $L^{2}$-error is similar to fixed regularization factor cases at the early inversion stage, but eventually becomes smaller as the inversion progresses.

### 4.2. Parametric Studies for Optimal EIT Result

**Figure 14:**
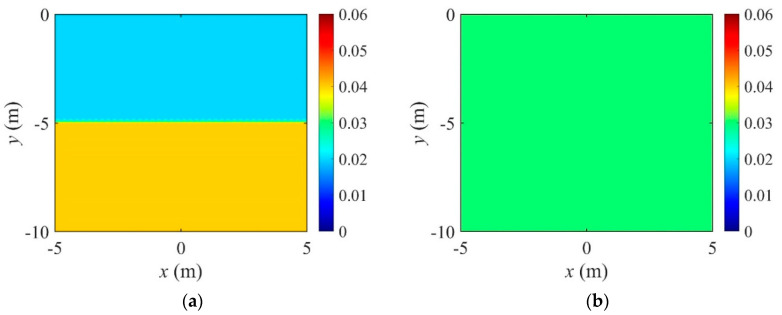
Target electrical conductivity profile with two layers and the initial guess for inversion. (**a**) Target. (**b**) Initial guess.

In this study, three different analysis conditions were explored to derive optimal parameters for performing the EIT in heterogeneous media. The conditions considered are the number of electrodes, spatial current input pattern, and electrode arrangement. Figure 14 shows the target electrical conductivity profile with two layers and the initial guess for inversion. The target values of the electrical conductivity are 0.02 S/m for y≥−5 m and 0.04 S/m for y<−5 m. The initial guess of the profile is homogeneous, with $\sigma_{\mathrm{ini}}=0.03$ S/m. The TV regularization with the regularization factor continuation scheme is used in all parametric studies.

#### 4.2.1. Number of Electrodes

**Figure 15:**
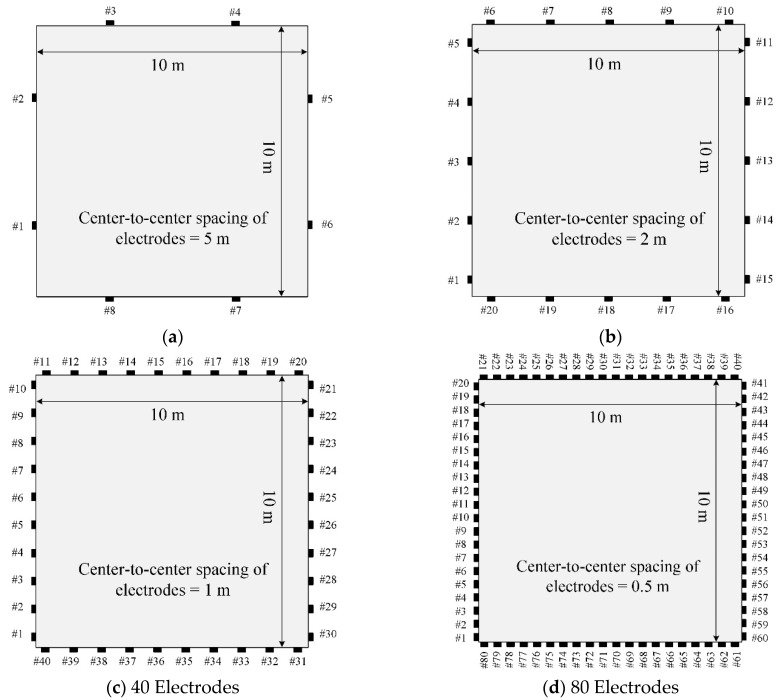
Different number of electrodes for the EIT of a heterogeneous square medium: source electrode, top and left electrodes; receiver electrode, right and bottom electrodes. (**a**) 8 Electrodes. (**b**) 20 Electrodes. (**c**) 40 Electrodes. (**d**) 80 Electrodes.

The number of electrodes is expected to significantly affect the inversion result because it impacts the electric potential field and the amount of measured potential data. In this work, 8, 20, 40, and 80 electrodes are tested for inversion, as shown in Figure 15. An equal number of electrodes are placed on each side of the square medium. The current is supplied into the electrodes attached to the top and left sides of the medium, and then flows out from the electrodes on the right and bottom sides. The magnitude of the current is uniform at 0.1 A.

**Figure 16:**
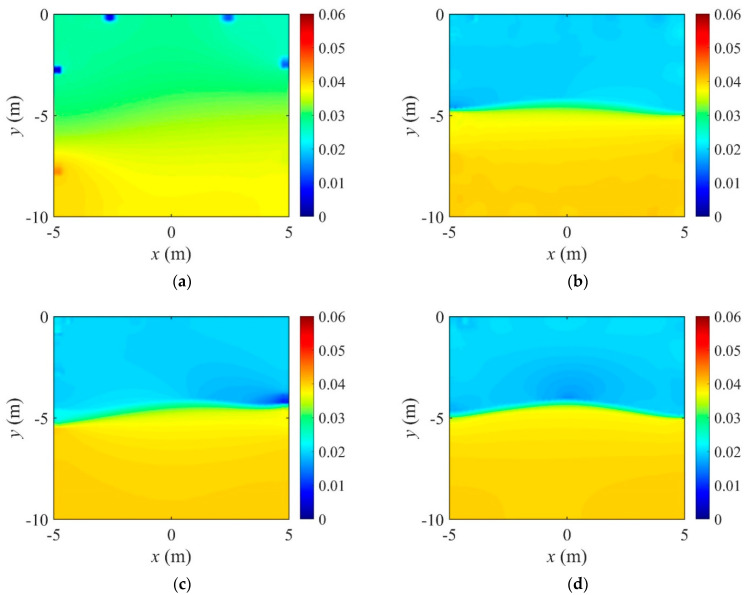
Reconstructed two-layer electrical conductivity profiles using different number of electrodes. (**a**) 8 Electrodes. (**b**) 20 Electrodes. (**c**) 40 Electrodes. (**d**) 80 Electrodes.

**Figure 17:**
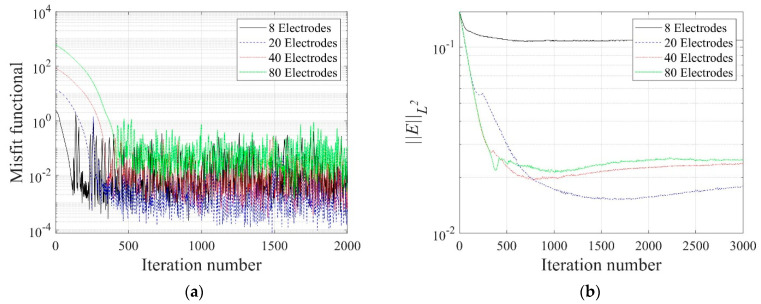
Variation of response misfit and the relative $L^{2}$-error to iteration numbers during the inversion using four different electrode numbers. (**a**) Misfit variation. (**b**) Relative $L^{2}$-error.

**Figure 18:**
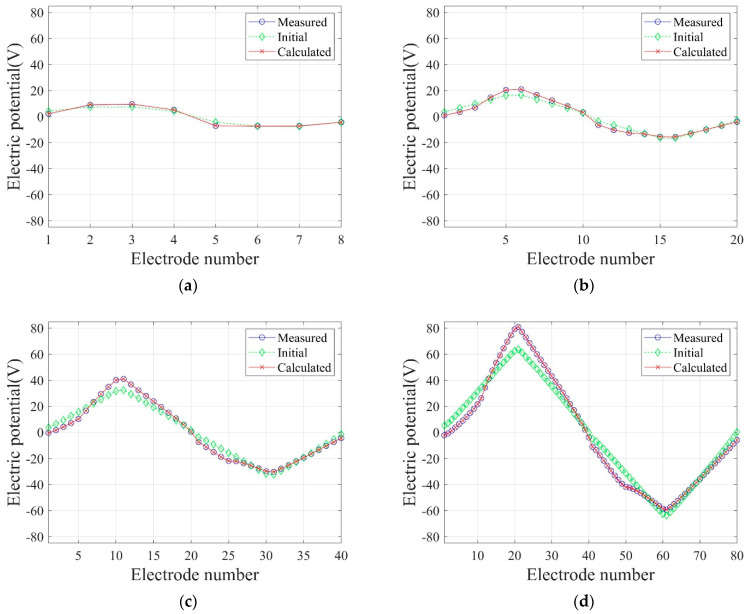
Measured, initial, and calculated electric potentials in the inversion using four different electrode numbers. (**a**) 8 Electrodes. (**b**) 20 Electrodes. (**c**) 40 Electrodes. (**d**) 80 Electrodes.

**Figure 19:**
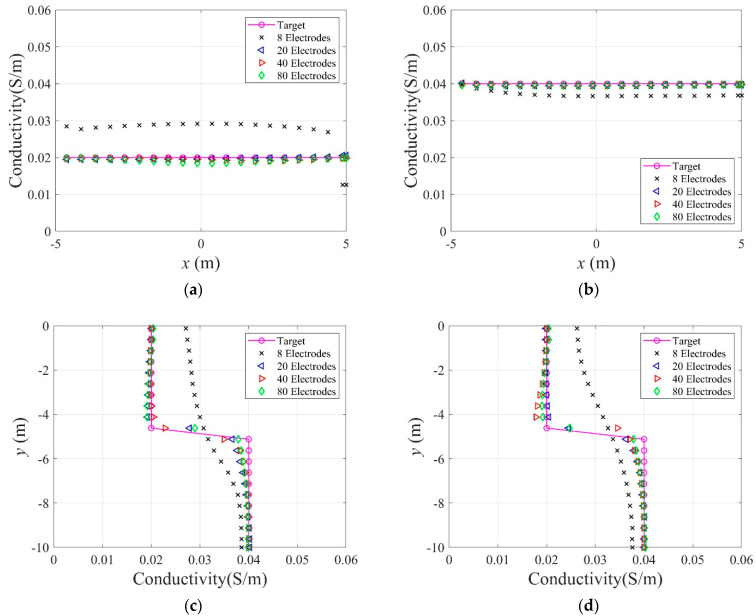
Reconstructed electrical conductivity profiles at several locations of the domain after the inversion using four different electrode numbers. (**a**)  y=−2.5 m. (**b**)  y=−7.5 m. (**c**) x=−3.0 m. (**d**)  x=3.0 m.

**Figure 20:**
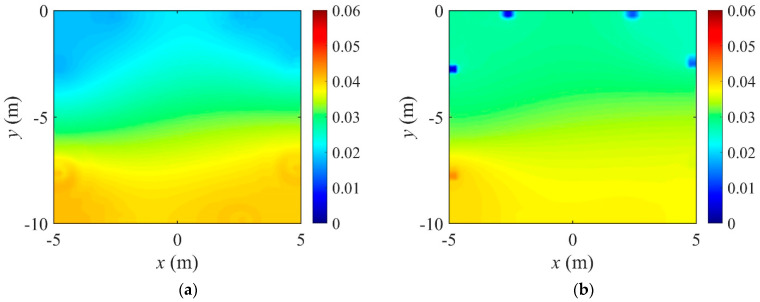
Inverted two-layer conductivity profiles using TN and TV regularizations in the case of 8 electrodes. (**a**) TN. (**b**) TV.

Figure 16 shows the reconstructed two-layer electrical conductivity profiles at 2000 inversion iterations using the presented number of electrodes. In the case of eight electrodes, the target profile is not reconstructed properly compared to other cases because the number of electrodes on the surface is significantly insufficient. In other cases, the target profile is reasonably reconstructed. Figure 17a shows the variation of response misfit to iteration numbers during the inversion. In the case of eight electrodes, the misfit ${ℱ}_{m}$ changes extremely unstable, even for small iteration numbers. In other cases, ${ℱ}_{m}$ decreases by a factor of $10^{-3}$ to $10^{-4}$. Figure 17b exhibits the variation of the relative $L^{2}$-error to iteration numbers. In the case of 20 electrodes, the error is smaller than 40 and 80 electrode cases after about 750 iterations. Thus, a higher number of electrodes does not necessarily increase the inversion quality. Figure 18 shows the measured, initial, and calculated electric potentials in the inversion using four different electrode numbers. After the inversion, the calculated potential values coincide with the measured values. Figure 19 shows the reconstructed electrical conductivity profiles at y=−7.5 m, y=−2.5 m, x=−3.0 m, and x=3.0 m after the inversion using four different electrode numbers. Except for the case of eight electrodes, the calculated conductivity values capture the target sufficiently well in all locations. Figure 20 presents the inverted profiles using TN and TV regularization schemes in the case of eight surface electrodes. In comparison with the TV scheme, the inversion result has been improved in the case of TN regularization. Therefore, it is more appropriate to use TN regularization when the number of electrodes is small.

#### 4.2.2. Current Input Pattern

Uniform and cosine input patterns are investigated as the current input pattern for inversion. The uniform pattern is the same as that used in Section 4.2.1. In the case of the cosine pattern, this study introduced four current input phases to the inversion. Equations (33) and (34) describe the uniform and cosine current input patterns, respectively.

(33) $$I_{l}=\{\begin{array}{c} 0.1\;A,\;\;1\leq l\leq \frac{L}{2}\\ -0.1\;A,\;\;\frac{L}{2}<l\leq L \end{array}\;\;\;\;\;\;\left(\mathrm{Uniform}\right),$$

(34) $$I_{l}=\cos\left(\frac{4\pi k}{N}-\alpha \right)A,\alpha =0,\;\frac{\pi }{4},\;\frac{\pi }{2},\;\frac{3\pi }{4}\left(\mathrm{Cos}\mathrm{ine}\right).$$

**Figure 21:**
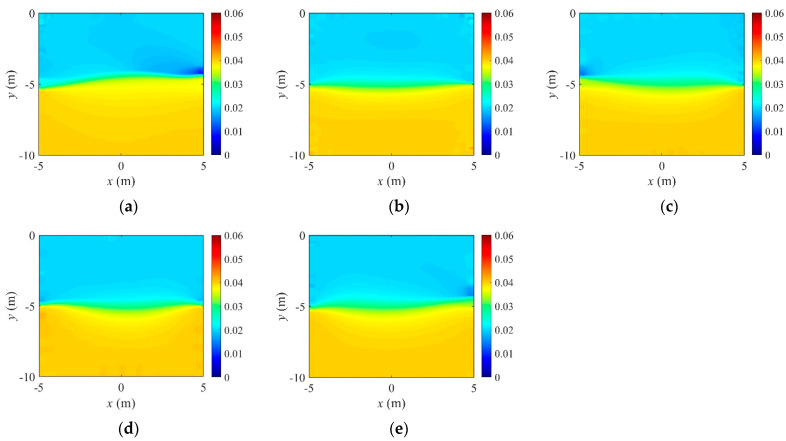
Reconstructed two-layer electrical conductivity profiles using uniform and cosine current input patterns. (**a**) Uniform. (**b**) Cosine, α=0. (**c**) Cosine, $\alpha =\frac{\pi }{4}$. (**d**) Cosine, $\alpha =\frac{\pi }{2}$. (**e**) Cosine, $\alpha =\frac{3\pi }{4}$.

**Figure 22:**
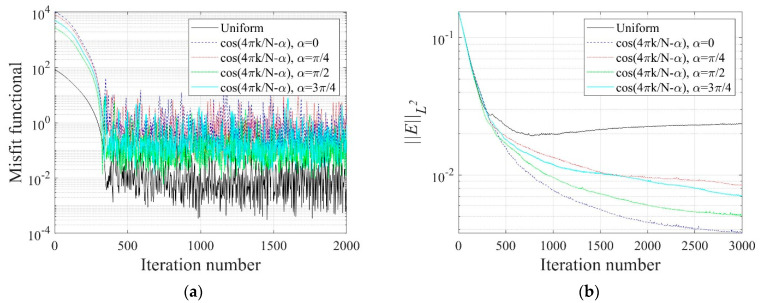
Variation of response misfit and the relative $L^{2}$-error to iteration numbers during the inversion using uniform and cosine current input patterns. (**a**) Misfit variation. (**b**) Relative $L^{2}$-error.

**Figure 23:**
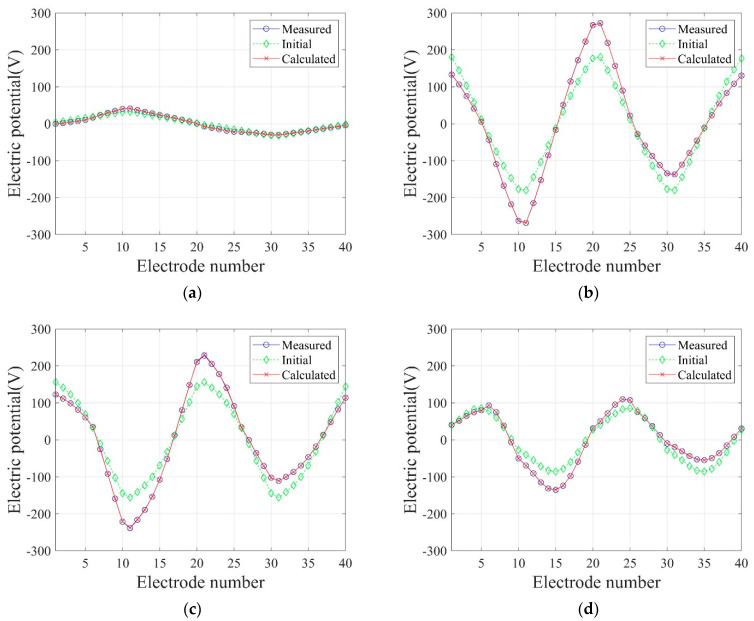

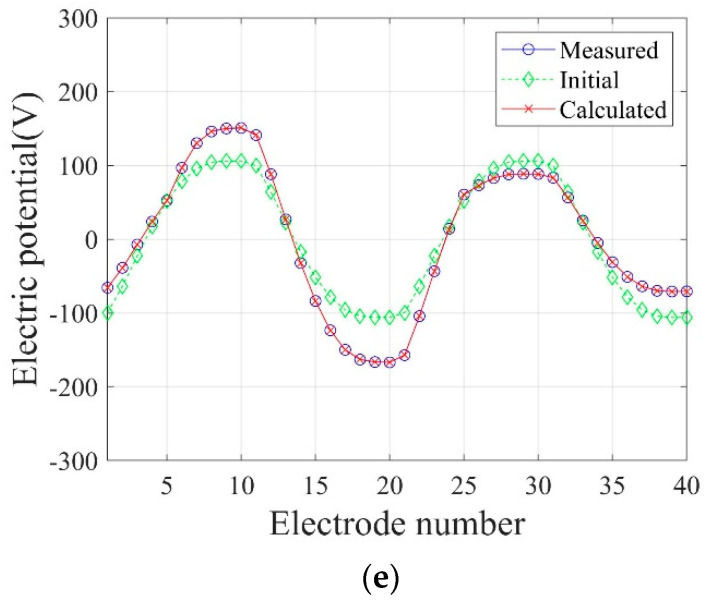
Measured, initial, and calculated electric potentials in the inversion using uniform and cosine current input patterns. (**a**) Uniform. (**b**) Cosine, α=0. (**c**) Cosine, $\alpha =\frac{\pi }{4}$. (**d**) Cosine, $\alpha =\frac{\pi }{2}$. (**e**) Cosine, $\alpha =\frac{3\pi }{4}$.

**Figure 24:**
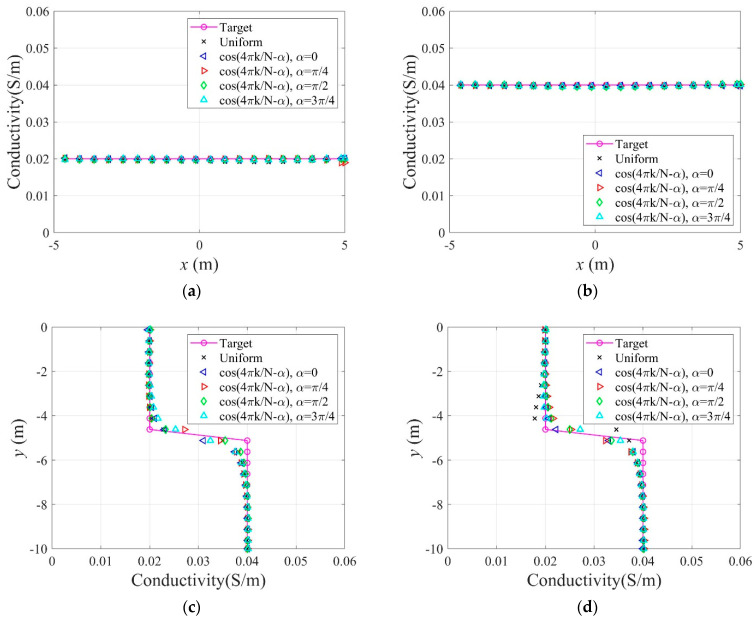
Reconstructed electrical conductivity profiles at several locations of the domain in the inversion using uniform and cosine current input patterns. (**a**)  y=−2.5 m. (**b**)  y=−7.5 m. (**c**) x=−3.0 m. (**d**)  x=3.0 m.

In Equation (34), N denotes the number of electrodes, and k is a specific electrode number. In this numerical experiment, N is 40, as shown in Figure 15c. Figure 21 shows the inverted two-layer electrical conductivity profiles at 2000 inversion iterations using the described current input patterns. Despite some differences in the results, especially at the layer interface, all current patterns successfully reconstructed the target profile. Figure 22 shows the response misfit and relative $L^{2}$-error to iteration numbers in the inversion using the current input patterns. In all cases, the misfit is reduced by more than 99.9% from the initial misfit at 500 iterations. Figure 22b shows that the relative $L^{2}$-error in the case of the uniform pattern is larger than that of the cosine patterns. The error is smallest when the phase α=0. Figure 23 shows the measured, initial, and calculated electric potentials in the inversion using the current input patterns. Again, the calculated potential values are almost identical to the measured values. Figure 24 shows the reconstructed conductivity profiles at y=−7.5 m, y=−2.5 m, x=−3.0 m, and x=3.0 m after the inversion using the current input patterns. All patterns reconstruct the target electrical conductivity profile fairly well.

#### 4.2.3. Electrode Arrangement

**Figure 25:**
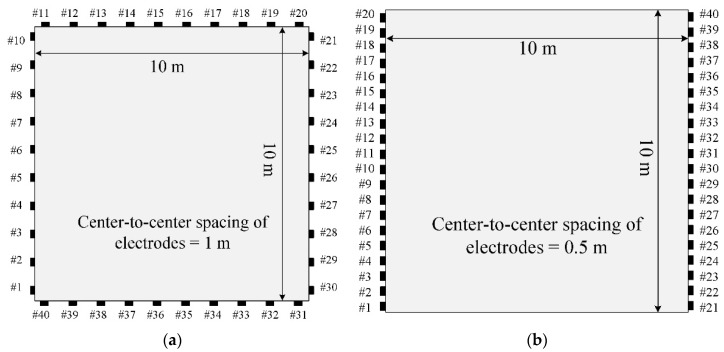
Configuration of two different types of electrode arrangement; For the uniform current input pattern, the source electrode is #1 to #20, and receiver electrode is #21 to #40. For the cosine input pattern with α=π/2, source and receiver electrodes are determined per Equation (34). (**a**) All-side. (**b**) Two-side.

Figure 25 shows two different types of electrode arrangements used for the EIT. The first arrangement type is to place electrodes on all sides of the medium, and the second is to position them only on two sides. The total number of electrodes is 40. The uniform and cosine ($\alpha =\frac{\pi }{2})$ current input patterns are used for this case.

**Figure 26:**
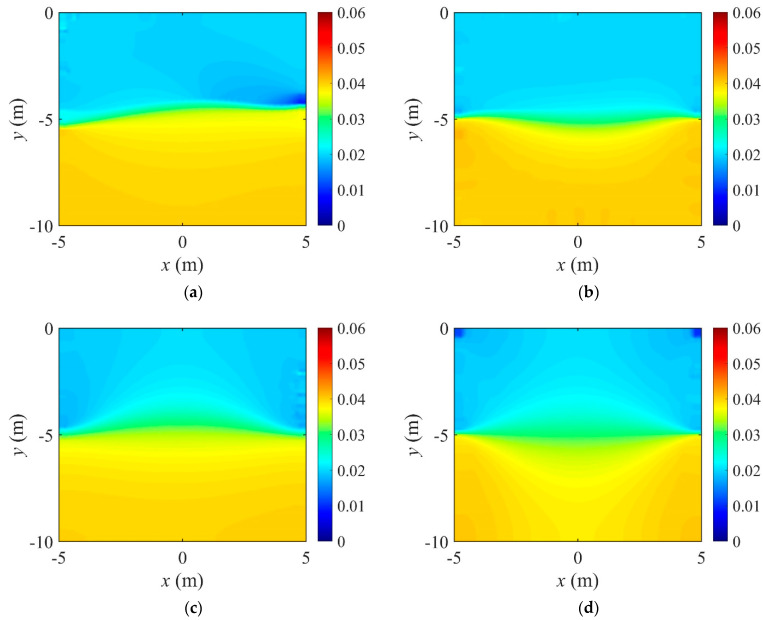
Reconstructed two-layer electrical conductivity profiles using two electrode arrangements. (**a**) All-side, uniform. (**b**) All-side, cosine, $\alpha =\frac{\pi }{2}$. (**c**) Two-side, uniform. (**d**) Two-side, cosine, $\alpha =\frac{\pi }{2}$.

**Figure 27:**
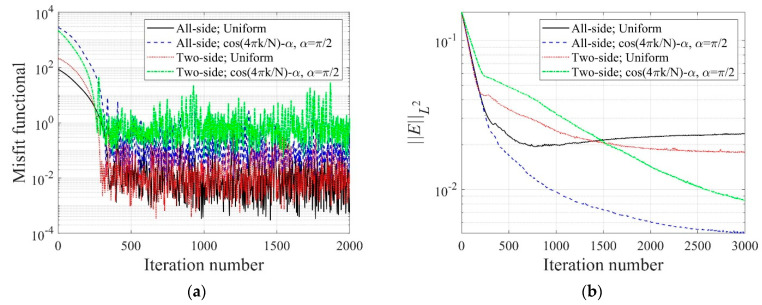
Variation of response misfit and the relative $L^{2}$-error to iteration numbers during the inversion using two electrode arrangements. (**a**) Misfit variation. (**b**) Relative $L^{2}$-error.

**Figure 28:**
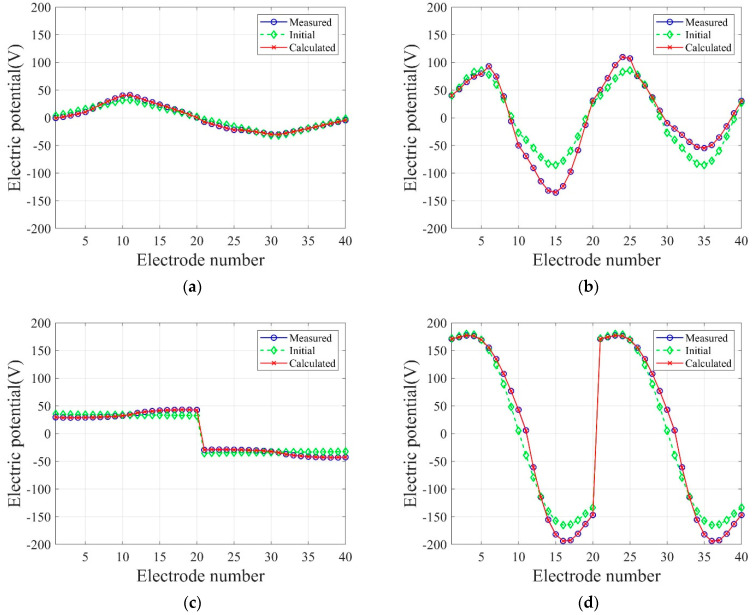
Measured, initial, and calculated electric potentials in the inversion using two electrode arrangements. (**a**) All-side, uniform. (**b**) All-side, cosine, $\alpha =\frac{\pi }{2}$. (**c**) Two-side, uniform. (**d**) Two-side, cosine, $\alpha =\frac{\pi }{2}$.

**Figure 29:**
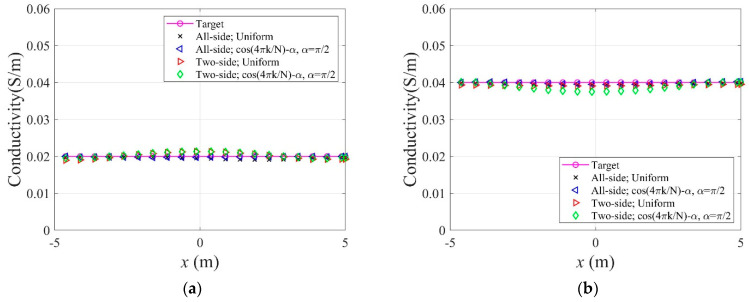

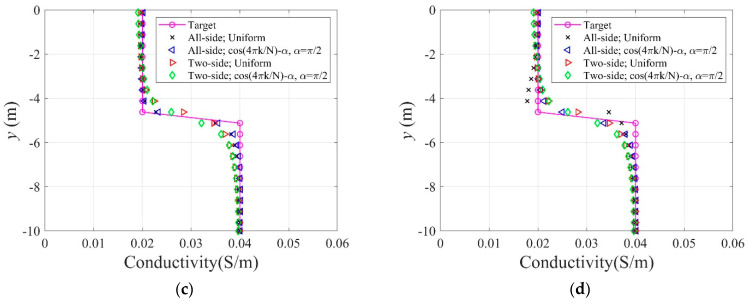
Reconstructed electrical conductivity profiles at several locations of the domain in the inversion using two electrode arrangements. (**a**)  y=−2.5 m. (**b**)  y=−7.5 m. (**c**) x=−3.0 m. (**d**)  x=3.0 m.

Figure 26 shows the inversion results at 2000 iterations using the two electrode arrangements. The target profile has been reconstructed well for both current input patterns. However, the quality of reconstruction at the layer interface is better for the all-side arrangement. This happens when the electrodes are attached to only two sides of the structure, and the inner information of the top and bottom parts cannot be sufficiently captured by surface electrodes. Figure 27 shows the response misfit and the relative $L^{2}$-error to iteration numbers during the inversion using the two electrode arrangements. For all cases, the misfit decreased by a factor of $10^{-3}$ to $10^{-4}$. As shown in Figure 27b, the relative $L^{2}$-error for the all-side arrangement is smaller than for the two-side arrangement. In addition, the error for the cosine current pattern is smaller than for the uniform pattern. Figure 28 shows the measured, initial, and calculated electric potentials in the inversion using the two electrode arrangements. The excellent agreement of the calculated and measured electric potential values demonstrates the feasibility of the inversion. Figure 29 presents the target and reconstructed electrical conductivity profiles at y=−7.5 m, y=−2.5 m, x=−3.0 m, and x=3.0 m. Overall, the all-side arrangement results in better reconstruction of the target profile than the two-side arrangement.

### 4.3. Optimal Choice of Implementation Parameters

**Figure 30:**
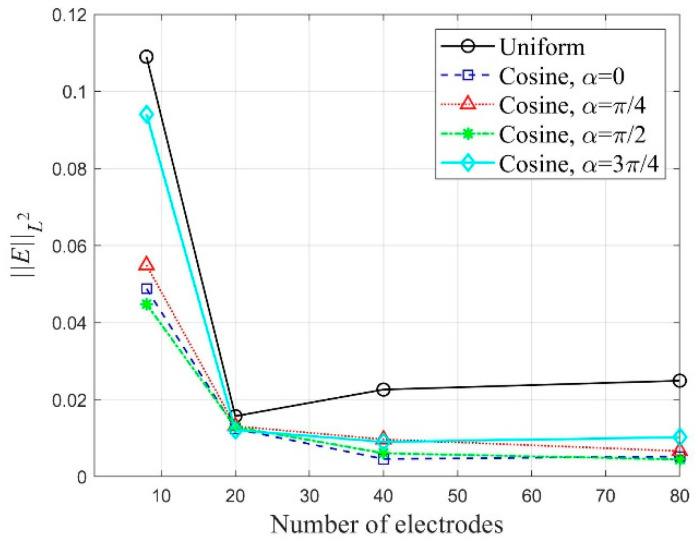
Variation of ${\left\|E\right\|}_{L^{2}}$ corresponding to the number of electrodes in the inversion using the all-side electrode arrangement; the ${\left\|E\right\|}_{L^{2}}$ values were calculated at 2000 inversion iterations.

**Figure 31:**
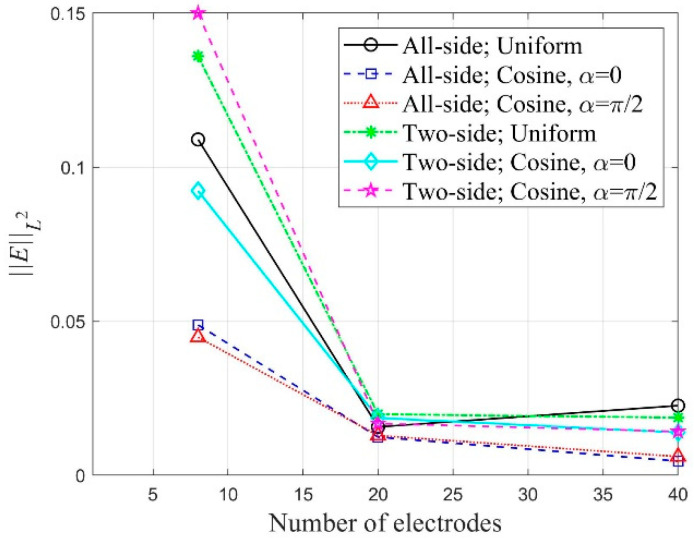
Variation of ${\left\|E\right\|}_{L^{2}}$ corresponding to the number of electrodes in the inversion cases of different electrode arrangement and current input pattern; the ${\left\|E\right\|}_{L^{2}}$ values were calculated at 2000 inversion iterations.

Figure 30 shows the variation of ${\left\|E\right\|}_{L^{2}}$ corresponding to the number of electrodes in the inversion using the all-side electrode arrangement. The values of ${\left\|E\right\|}_{L^{2}}$ were calculated after 2000 inversion iterations. As the number of electrodes increases, the relative $L^{2}$-error tends to decrease. However, when the number of electrodes is 40 or more, there is a slight difference in the error. In addition, the error reduces when the cosine current input pattern is used, especially when α=0 or α=π/2. A similar error trend can be observed in the case of the two-side electrode arrangement. Figure 31 presents the variation of ${\left\|E\right\|}_{L^{2}}$ corresponding to the number of electrodes in the inversion cases of different electrode arrangement and current input pattern. The relative $L^{2}$-error is smaller in the case of the all-side electrode arrangement than in the two-side arrangement.

Table 2 and Table 3 show the relative $L^{2}$-error and the relative misfit (${\left|{ℱ}_{m}\right|}_{\mathrm{opt}}/{\left|{ℱ}_{m}\right|}_{\mathrm{ini}}$) for all cases of the number of electrodes, current input pattern, and electrode arrangement discussed so far. The misfit, ${\left|{ℱ}_{m}\right|}_{\mathrm{opt}}$, is the one immediately before the start of the misfit oscillation, and ${\left|{ℱ}_{m}\right|}_{\mathrm{ini}}$ is the initial misfit. From the error values in the tables, one can choose the optimal implementation parameters of the described EIT. The first parameter set for which the value of ${\left\|E\right\|}_{L^{2}}$ is minimal is 80 electrodes, cosine current input pattern with α=π/2, and the all-side electrode arrangement. The second parameter set for which the value of ${\left|{ℱ}_{m}\right|}_{\mathrm{opt}}/{\left|{ℱ}_{m}\right|}_{\mathrm{ini}}$ is minimal is the same as the first set except for the number of electrodes, which is 40. The minimum error values are shaded in the tables.

**Figure 32:**
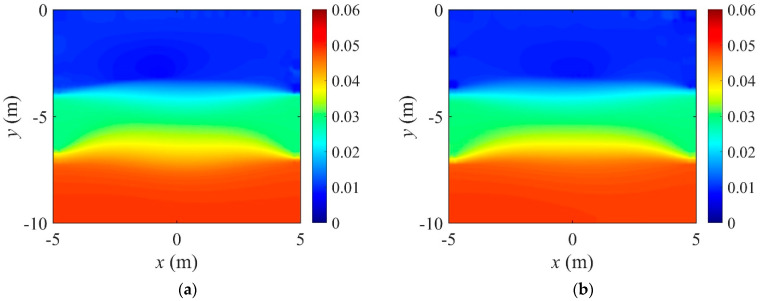
Reconstructed three-layer electrical conductivity profiles using the optimal implementation parameters. (**a**) First parameter set. (**b**) Second parameter set.

**Figure 33:**
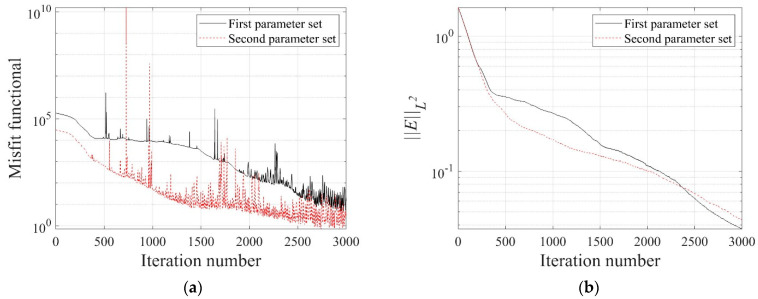
Response misfit and relative $L^{2}$-error against iteration numbers in the inversion using the optimal implementation parameters. (**a**) Misfit variation. (**b**) Relative $L^{2}$-error.

Figure 32 shows the reconstructed three-layer profiles using the optimal implementation parameters. The target and initial guess of the electrical conductivity profile are the same as those in Figure 5. The inverted profiles are obtained at 3000 iterations. The quality of profile reconstruction using the optimal parameter set is considerably better than the result shown in Figure 6, especially at the layer interface. Figure 33 shows the misfit and the relative $L^{2}$-error against iteration numbers in the inversion using the optimal parameter sets. The relative $L^{2}$-error decreased by 95.1% compared to the initial value when the first parameter set is used. It also decreased by 94.4% when using the second parameter set. The reduction rate of ${\left\|E\right\|}_{L^{2}}$ is slightly lower than in Figure 7b.

## 5. Conclusions

This study investigated optimal implementation parameters for a nonlinear EIT technique using the CEM. The EIT method is based on PDE-constrained optimization, which reconstructs the electrical conductivity profile by solving the KKT conditions iteratively. By applying various analysis conditions, the optimal set of parameters that minimize relative $L^{2}$-error or relative misfit in the EIT has been derived. The quality of the reconstructed profile using the optimal implementation parameters is superior to the results using a conventional parameter set.

1. The layered profile was reconstructed more clearly when using the TV regularization scheme than TN, especially at the interface of layers. The inversion result was improved when using the regularization factor continuation scheme rather than the fixed method.
2. A higher number of electrodes did not necessarily improve the inversion results. In addition, the TN regularization scheme produced relevant results when the number of electrodes was small.
3. The layered profiles were successfully reconstructed for all the presented current patterns. The relative $L^{2}$-error was smaller when the cosine pattern was used, especially when the phase α=0 or $\alpha =\frac{\pi }{2}$.
4. In the case of arranging electrodes on all sides of the square domain, the inversion result was improved compared to the case of arranging them only on two sides.
5. The relative $L^{2}$-error and the relative misfit are proper criteria for optimal implementation parameters. The relative $L^{2}$-error was decreased by 95.1% from the initial value when using the first set of optimal parameters. It was also reduced by 94.4% when using the second set. The presented optimal parameter sets worked successfully in reconstructing layered electrical conductivity profiles.

This study is expected to expand the applicability of the nonlinear EIT method for the non-destructive evaluation of civil structures such as damage inspection, strength inspection of concrete under curing, fiber content inspection of fiber-reinforced concrete, etc.

## Figures and Tables

**Table 1:** Backtracking algorithm to determine the step length α.

Choose $\bar{\alpha }>0$, $\bar{\alpha }$, $\bar{\mu }\in \left(0,\;1\right);$ set $\alpha \leftarrow \bar{\alpha };$ repeat $\alpha \leftarrow \bar{\rho }\alpha ;$ until $J\left(\sigma_{k}+\alpha d_{k}\right)\leq J\left(\sigma_{k}\right)+\bar{\mu }\alpha g_{k}\cdot d_{k}$ Terminate with $\alpha_{k}=\alpha$

**Table 2:** Relative $L^{2}$-error, ${\left\|E\right\|}_{L^{2}}$, for all the inversion cases of this study.

Current Input Pattern	Electrode Arrangement							
	All-Side Arrangement				Two-Side Arrangement			
	Number of Electrodes				Number of Electrodes			
	8	20	40	80	8	20	40	80
Uniform	$1.09\times 10^{-1}$	$1.57\times 10^{-2}$	$2.26\times 10^{-2}$	$2.49\times 10^{-2}$	$1.36\times 10^{-1}$	$1.98\times 10^{-2}$	$1.87\times 10^{-2}$	$2.72\times 10^{0}$
Cosine, α=0	$4.88\times 10^{-2}$	$1.24\times 10^{-2}$	$4.58\times 10^{-3}$	$5.21\times 10^{-3}$	$9.23\times 10^{-2}$	$1.86\times 10^{-2}$	$1.39\times 10^{-2}$	$3.58\times 10^{0}$
Cosine, $\alpha =\frac{\pi }{4}$	$5.49\times 10^{-2}$	$1.31\times 10^{-2}$	$9.66\times 10^{-3}$	$6.63\times 10^{-3}$	$1.18\times 10^{-1}$	$4.77\times 10^{-2}$	$2.62\times 10^{-2}$	$4.24\times 10^{0}$
Cosine, $\alpha =\frac{\pi }{2}$	$4.48\times 10^{-2}$	$1.29\times 10^{-2}$	$6.07\times 10^{-3}$	$4.45\times 10^{-3}$	$1.50\times 10^{-1}$	$1.68\times 10^{-2}$	$1.42\times 10^{-2}$	$5.26\times 10^{0}$
Cosine, $\alpha =\frac{3\pi }{4}$	$9.41\times 10^{-2}$	$1.21\times 10^{-2}$	$9.02\times 10^{-3}$	$1.02\times 10^{-2}$	$9.52\times 10^{-2}$	$3.11\times 10^{-2}$	$3.04\times 10^{-2}$	$2.88\times 10^{0}$

**Table 3:** Relative misfit, ${\left|{ℱ}_{m}\right|}_{\mathrm{opt}}/{\left|{ℱ}_{m}\right|}_{\mathrm{ini}}$, for all the inversion cases of this study.

Current Input Pattern	Electrode Arrangement							
	All-Side Arrangement				Two-Side Arrangement			
	Number of Electrodes				Number of Electrodes			
	8	20	40	80	8	20	40	80
Uniform	$1.21\times 10^{-3}$	$3.55\times 10^{-5}$	$4.37\times 10^{-5}$	$2.82\times 10^{-5}$	$3.42\times 10^{-4}$	$5.05\times 10^{-6}$	$3.01\times 10^{-5}$	$3.36\times 10^{-5}$
Cosine, α=0	$5.22\times 10^{-3}$	$7.53\times 10^{-5}$	$4.94\times 10^{-5}$	$1.20\times 10^{-4}$	$1.26\times 10^{-2}$	$5.45\times 10^{-5}$	$1.26\times 10^{-4}$	$1.37\times 10^{-4}$
Cosine, $\alpha =\frac{\pi }{4}$	$1.28\times 10^{-4}$	$9.16\times 10^{-5}$	$3.01\times 10^{-5}$	$1.69\times 10^{-4}$	$7.10\times 10^{-4}$	$2.57\times 10^{-5}$	$7.58\times 10^{-6}$	$2.12\times 10^{-4}$
Cosine, $\alpha =\frac{\pi }{2}$	$9.46\times 10^{-3}$	$1.37\times 10^{-4}$	$4.84\times 10^{-6}$	$3.87\times 10^{-5}$	$8.05\times 10^{-4}$	$1.02\times 10^{-4}$	$2.00\times 10^{-4}$	$4.75\times 10^{-4}$
Cosine, $\alpha =\frac{3\pi }{4}$	$4.94\times 10^{-5}$	$2.87\times 10^{-4}$	$8.43\times 10^{-6}$	$1.33\times 10^{-4}$	$7.18\times 10^{-5}$	$2.80\times 10^{-4}$	$3.01\times 10^{-5}$	$1.23\times 10^{-4}$

## Data Availability

The data that support the findings of this study are available from the corresponding author, Jun Won Kang, upon reasonable request.

## Nomenclature

Alphabetic symbols	
A	Area of square domain
$d_{k}$	Search direction vector
$E_{l}$	lth electrode
$e_{l}$	Length of lth electrode
F	Force vector
$g_{k}$	Discrete reduced gradient at kth inversion iteration
H	Hessian matrix
$I_{l}$	Magnitude of current injected at lth electrode
J	Current density
K	Stiffness matrix
L	Number of electrodes
N	Number of nodes in finite element mesh
$n_{j}$	Basis vector
$R_{\sigma }$	Regularization factor
U	Vector consisting of electric potential at each electrode
$U_{l}$	Electric potential at lth electrode
$U_{l}^{m}$	Measured electric potential at lth electrode
u	Solution vector
u	Electric potential in domain
$V_{l}$	Test value
v	Test function
$W_{l}$	Lagrange multiplier
w	Lagrange multiplier
$z_{l}$	Contact impedance at lth electrode
Greek symbols	
α	Step length
$\alpha_{j}$	Nodal value of u(x)
$\bar{\alpha }$	Initial step length
$\alpha_{j}^{\mathrm{adj}}$	Nodal value of w(x)
β	Small parameter for TV regularization scheme
$\beta_{j}$	$\mathrm{Unknown}\;\mathrm{parameter}\;\mathrm{for}\;n_{j}$ $\;\mathrm{and}\;U^{h}$
$\beta_{j}^{\mathrm{adj}}$	$\mathrm{Unknown}\;\mathrm{parameter}\;\mathrm{for}\;n_{j}$ $\;\mathrm{and}\;W^{h}$
$\Gamma_{E_{l}}$	Boundary of lth electrode
ε	$\mathrm{Weight}\;\mathrm{factor}\;\mathrm{of}\;R_{\sigma }$
$\bar{\mu }$	Small parameter for Armijo condition
$\bar{\rho }$	Parameter for reducing step length
σ	Electrical conductivity
$\phi_{i}$	Legendre basis function
Ω	Structural domain
